# Predictive and prognostic role of tumour-infiltrating lymphocytes in breast cancer patients with different molecular subtypes: a meta-analysis

**DOI:** 10.1186/s12885-020-07654-y

**Published:** 2020-11-25

**Authors:** Zhao-hua Gao, Cun-xin Li, Ming Liu, Jia-yuan Jiang

**Affiliations:** 1 Department of Breast Surgery, Cancer Hospital of China Medical University, No.44 Xiaoheyan Road, Dadong District, Shenyang, 110042 Liaoning Province P.R. China; 2 Department of Breast Surgery, Liaoning Cancer Hospital & Institute, No.44 Xiaoheyan Road, Dadong District, Shenyang, 110042 Liaoning Province P.R. China; 3 Department of Breast Surgery, Dalian Medical University Clinical Oncology College, Shenyang, Liaoning 110042 P.R. China

**Keywords:** Breast cancer, Tumour-infiltrating lymphocytes, Molecular subtype, Prognosis, Prediction, Meta-analysis

## Abstract

**Background:**

Whether tumour-infiltrating lymphocytes (TILs) play different roles in different molecular subtypes of breast cancer remains unknown. Additionally, their prognostic and predictive value in different molecular subtypes of breast cancer is still controversial. The aim of our meta-analysis was to assess the prognostic and predictive value of TILs in different molecular subtypes of breast cancer by summarizing all relevant studies performing multivariate analysis.

**Methods:**

PubMed, Embase, EBSCO, ScienceDirect, the Cochrane Database and Web of Science were comprehensively searched (until March 2020). Hazard ratios (HRs), odds ratios (ORs) and their 95% confidence intervals (CIs) were used as effect measures to perform our meta-analysis. A random effect model was used. Stata software, version 15 (2017) (StataCorp, College Station, TX, USA) was used to perform the statistical analysis.

**Results:**

Thirty-three studies including 18,170 eligible breast cancer patients were analysed. The meta-analysis showed that high TIL expression was significantly associated with increased pathological complete response (pCR) rates after neoadjuvant chemotherapy in patients with the HER2-enriched molecular subtype (OR = 1.137, 95% CI [1.061 ~ 1.218], *p* < 0.001) and triple-negative breast cancer (TNBC) subtype (OR = 1.120, 95% CI [1.061 ~ 1.182], *p <* 0.001). However, high TIL expression was not significantly associated with high pCR rates after neoadjuvant chemotherapy in patients with the luminal molecular subtype of breast cancer (OR = 1.154, 95% CI [0.789 ~ 1.690], *p* = 0.460). We carried out a meta-analysis on the HRs of overall survival (OS) and disease-free survival (DFS) to assess the prognostic value of TILs in breast cancer with different molecular subtypes more deeply. Our meta-analysis confirmed that high TILs were associated with significantly improved DFS in patients with the HER2-enriched molecular subtype [HR = 0.940, 95% CI (0.903 ~ 0.979), *p* = 0.003] and TNBC molecular subtype [HR = 0.907, 95% CI (0.862 ~ 0.954), *p* < 0.001]. However, high TILs were not associated with significantly better DFS in patients with the luminal molecular subtype of breast cancer [HR = 0.998, 95% CI (0.977 ~ 1.019), *p* = 0.840]. Furthermore, the results confirmed that high TILs were significantly related to better OS in patients with the HER2-enriched molecular subtype [HR = 0.910, 95% CI (0.866 ~ 0.957), *p* < 0.001] and TNBC molecular subtype [HR = 0.869, 95% CI (0.836 ~ 0.904), *p* < 0.001]. Conversely, the summarized results indicated that high TILs were significantly associated with poor OS in patients with the luminal molecular subtype of breast cancer [HR = 1.077, 95% CI (1.016 ~ 1.141), *p* = 0.012].

**Conclusions:**

Our meta-analysis confirms that high TILs are associated with favourable survival and predicts pCR in breast cancer patients with the TNBC and HER2-enriched molecular subtypes.

## Background

Breast cancer is one of the most common malignant tumours in women [1] and is still the second leading cause of cancer-related death in women around the world [2]. At present, the forecast of prognosis is not ideal, and a specific predictor is needed to enhance the individualized therapeutic effect. The complex interaction between the immune system and cancer cells plays a vital role in controlling and eradicating cancer and is regulated by a delicate balance between activation and suppression signals [3]. Research on the microenvironment of tumours can reveal the complex correlation between the immune system and the biological behaviour of cancer cells. To restrict the development of breast cancer, it is very important to understand the tumour microenvironment.

Increasing evidence indicates that the tumour microenvironment plays an important role in tumour formation, growth, invasion and metastasis. Tumour-infiltrating lymphocytes (TILs) have emerged as potentially important prognostic and/or predictive biomarkers for breast cancer [4, 5]. Although valuable information has been obtained, the heterogeneity in experimental design and TIL assessment has hindered a more comprehensive understanding of the biological value of TILs. However, the prognostic value of TIL remains complex and controversial. Breast cancer is a clinically and molecularly heterogeneous disease, and various factors determine the prognosis and response to treatment.

Thus, we carried out this meta-analysis, aiming to estimate the prognostic and predictive value of TILs in patients with different molecular subtypes of breast cancer.

## Methods

### Retrieval strategy

Embase, PubMed, EBSCO, the Cochrane Database, ScienceDirect and Web of Science were comprehensively searched for studies exploring the prognostic and predictive relationship between TILs and the different subtypes of breast cancer (without time, place of publication or language restrictions; until March 2020). No retrieval restrictions were used. In addition, the reference lists of searched reviews and studies were examined to further identify potentially related articles. The main retrieval terms applied were “breast cancer” or “breast carcinoma” and “neoadjuvant chemotherapy” and “TILs” or “Tumor-infiltrating lymphocytes” and “prognosis” or “change”.

### Selection standards

To ensure the accuracy and reliability of our analysis, we selected qualified studies based on the following criteria. (i) The prognostic or predictive value of TIL testing in different subtypes of breast cancer with at least one relevant outcome indicator was reported in the research or could be computed based on published data. (ii) The studies were of high quality and performed multivariate analysis on pathological complete response (pCR) or survival data such as disease-free survival (DFS) or overall survival (OS). (iii) The hazard ratio (HR), odds ratio (OR) and their 95% confidence intervals (CIs) were reported or could be calculated according to the outcome data (DFS, OS or pCR). (iv) The samples were taken from core-needle biopsy specimens or surgical specimens after the operation.

Two authors (Zhao-hua Gao and Ming Liu) independently performed the literature retrieval and determined qualified studies according to the inclusion criteria. Any disagreements between the authors were settled by discussion and consensus. If no agreement could be reached, the final outcome was determined by a third-party researcher (Cun-xin Li). If there was more than one publication on the basis of the same patient groups, the most informational research was used.

### Research quality appraisal and data collection

The data were collected according to the Cochrane guidelines [6]. Two authors (Zhao-hua Gao and Ming Liu) examined the eligible studies independently, and any disagreements between the authors were settled by discussion and consensus. The following data were collected for our meta-analysis: publication time, first author, country, study design, baseline patient characteristics, age range, treatment type, molecular subtypes, ethnicity, follow-up duration, TIL cut-off value, TIL position, outcomes (pCR, DFS, or OS), HR, OR and 95% CI. The Newcastle-Ottawa scale (NOS) criteria were used to evaluate the quality of the selected eligible studies [7]. A funnel plot was used to estimate the publication bias. The studies selected in our meta-analysis obtained written informed consent from all patients and were carried out according to clinical practice principles, all local regulations and the Helsinki Declaration.

### Statistical analysis

In this meta-analysis, we chose pCR as a predictor of neoadjuvant chemotherapy (NAC) for breast cancer. We assessed the overall OR and its 95% CI of the qualified studies to analyse the predictive value of TILs for NAC in breast cancer. OS and DFS were used as prognostic outcomes in our meta-analysis. In the meta-analysis, the HR and its 95% CI were used as the effect scales of prognosis. The associations between TILs and clinicopathological parameters were evaluated using ORs and 95% CIs. If the HR or OR and its 95% CI could not be obtained directly from the original article, we used the available data to calculate them with the software designed by Tierney et al. [8]. The Q test was used to estimate the heterogeneity between studies, and the I^2^ value represents the size of the heterogeneity [9]. I^2^ values > 40% indicated high heterogeneity [6]. If the heterogeneity was high, a random effect model was used; if not, a fixed effect model was used. The *P* value was set as < 0.05 to indicate statistical significance. The clinicopathological parameters and predictive and prognostic indicators of all relevant studies were pooled and analysed. At the same time, subgroup analysis was completed based on different countries and different study designs. The quality and homogeneity of the results were assessed by sensitivity analysis. A funnel plot was used to test publication bias. In the statistical analysis, we referred to the statistical parameters and methods used by our team in previous studies [10].

Stata software, version 15 (2017) (StataCorp, College Station, TX, USA) was used to carry out the statistical analysis. This meta-analysis followed the recommendations of the Preferred Reporting Items for Systematic Reviews and Meta-Analyses (PRISMA) guidelines [11].

## Results

### Baseline characteristics of the qualified studies

**Fig. 1:**
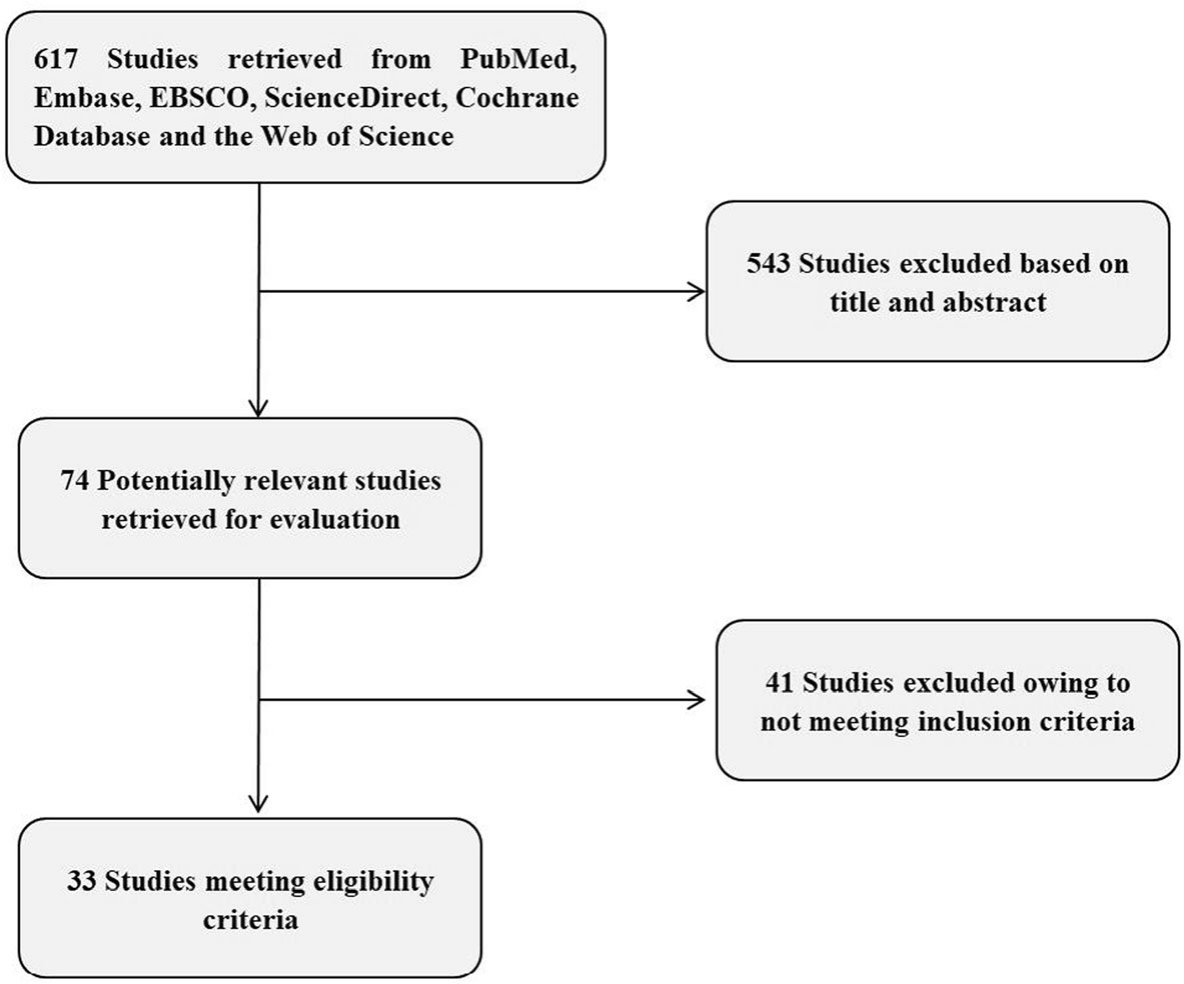
Selection process of included studies

In the systematic literature retrieval, we found 617 studies. By reviewing the titles and abstracts, 74 possible related studies were identified. Of these 74 studies, 41 studies were later excluded because they did not meet the selection criteria. Eventually, we determined that 33 studies met the inclusion criteria [4, 5, 12–42]. Fig. 1 summarizes the search and screening process. The 33 studies comprised 18,170 qualified patients with breast cancer (sample capacity, median: 331 [50–3771], mean: 550). These studies were published between 2010 and 2020 and were from Europe, America, Australia and Asia (Spain, France, Italy, United Kingdom, Belgium, Finland, Germany, Ireland, USA, Canada, Australia, Japan, Korea, and China). The eligible studies evaluated TILs by haematoxylin and eosin–stained sections. Twelve studies provided ORs for pCR to complete the meta-analysis [14, 24, 26, 27, 29, 32, 34–36, 38, 39, 42]. Fifteen of these studies provided HR data for DFS or OS, and we performed the pooled analysis. Twelve studies provided HR data for DFS [5, 14, 16–18, 25, 27, 30–32, 38, 41], and ten studies provided HR data for OS [5, 14, 16, 18, 19, 21, 22, 25, 27, 30]. Table 1 summarizes the main baseline characteristics. We evaluated the quality of the selected studies based on the NOS, as shown in Table 2.

**Table 1:** Baseline characteristics of included studies

first author	year of publication	Country	Study design	Number (n)	Treatment type	sample time	TILs site	TIL evaluation method	curative resection	Endpoint measured	Follow up Median (range)(M)
Hwang, Hye Won [11]	2019	Korea	retrospective	308	NAC	pre-NAC/post-NAC	sTILs	HE	YES	pCR/DFS/BCSS	60.1
Ahn, S. G [12].	2018	Korea	retrospective	198	not NAC	resection tissue	sTILs	HE	NR	NO	NR
Yang, Xia [13]	2018	China	retrospective	143	NAC + H	pre-NAC/post-NAC	sTILs	HE	YES	pCR/DFS/OS	53 (12–102)
Herrero-Vicent, C [14].	2017	Spain	retrospective	164	NAC	pre-NAC/post-NAC	sTILs	HE	NR	pCR/DFS	78
Luen, S. J [15].	2019	Australia	retrospective	375	NAC	pre-NAC/post-NAC	sTILs	HE	NR	RFS/OS	72
Fujimoto, Yukie [16]	2019	Japan	retrospective	717	NAC/adjuvant	pre-NAC/post-NAC	iTILs+sTILs	HE	YES	DFS/OS	35.1 (1–100.6)
Adams, S [17].	2014	USA	RCT	481	adjuvant	resection tissue	iTILs+sTILs	HE	NR	DFS/OS/DRFI	127.2
Dieci, M. V [18].	2014	France/Italy	retrospective	278	NAC/adjuvant	post-NAC	iTILs+sTILs	HE	NR	MFS/OS	75.6
Perez, E. A [19].	2016	USA	RCT	945	adjuvant	resection tissue	sTILs	HE	NR	RFS	52.8
Dieci, M. V [20].	2015	France	RCT	781	adjuvant	resection tissue	iTILs+sTILs	HE	NR	OS/DFS	152.4
Loi, S [5].	2013	Belgium	RCT	2009	adjuvant	resection tissue	iTILs+sTILs	HE	NR	DFS/OS	96
Loi, S [21].	2014	Finnish	retrospective	934	adjuvant	resection tissue	sTILs	HE	NR	DDFS/OS	62
Yasmin Issa-Nummer [22]	2013	Germany	RCT	313	NAC	pre-NAC	iTILs+sTILs	HE	NR	pCR	NR
Denkert, C [4].	2010	Germany	RCT	1058	NAC	pre-NAC	iTILs+sTILs	HE	NR	pCR	NR
Denkert, C [23].	2015	Germany	RCT	580	NAC	pre-NAC	iTILs+sTILs	HE	NR	pCR	NR
Pruneri, G [24].	2016	Italy	RCT	647	adjuvant	resection tissue	sTILs	HE	NR	BCFI / DFS / DRFI / OS	82.8
Ingold Heppner, B [25].	2016	Germany	RCT	498	NAC	pre-NAC	sTILs	HE	NR	pCR / DFS	60.4 (59.5–61.3)
Denkert, C [26].	2018	Germany	RCT	3771	NAC	pre-NAC	sTILs	HE	NR	pCR / DFS / OS	62.8
Wang, Qiong [27]	2020	China	retrospective	75	NAC	pre-NAC/post-NAC	sTILs	HE/IHC	YES	pCR / DFS	23.2 (6.1–64.5)
Brodsky, Alexander S [28].	2016	USA	retrospective	50	NAC + H	pre-NAC	sTILs	HE	NR	pCR	127.2
Leon-Ferre, Roberto A [29].	2018	USA	retrospective	605	adjuvant	resection tissue	iTILs+sTILs	HE	YES	IDFS / OS	127.2
Salgado, Roberto [30]	2015	Australia	RCT	387	NAC + H/L	pre-NAC	sTILs	HE	NR	EFS/ pCR	45.2 (42–50.6)
Ignatiadis, Michail [31]	2019	Belgium	RCT	213	NAC	pre-NAC/post-NAC	sTILs	HE	NR	pCR / EFS	56.4
Mori, H [32].	2017	Japan	retrospective	248	adjuvant	resection tissue	sTILs	HE	YES	RFS / OS	68 (2–150)
Dieci, M. V [33].	2016	Italy	retrospective	105	NAC	pre-NAC/post-NAC	iTILs+sTILs	HE	YES	pCR / EFS	NR
Ruan, Miao [34]	2018	China	retrospective	166	NAC	pre-NAC	iTILs+sTILs	HE	YES	pCR	NR
O’Loughlin, Mark [35]	2018	Ireland	retrospective	75	NAC	pre-NAC	sTILs	HE	NR	pCR	NR
Ali, H. Raza [36]	2016	UK	RCT	614	NAC	pre-NAC/post-NAC	sTILs	HE	NR	pCR	NR
Song, I. H [37].	2017	Korea	retrospective	108	NAC	pre-NAC/post-NAC	sTILs	HE/IHC	YES	pCR/DFS	31.4 (21.1–53.0)
Li, X [38].	2016	USA	retrospective	129	NAC + H	pre-NAC	iTILs+sTILs	HE	YES	pCR	NR
Würfel, F [39].	2018	Germany	retrospective	146	NAC	pre-NAC	sTILs	HE	NR	pCR	NR
Hamy, A. S [40].	2019	France	retrospective	718	NAC ± H	pre-NAC/post-NAC	sTILs	HE	YES	pCR/DFS/OS	NR
Khoury, T [41].	2018	USA	retrospective	331	NAC	pre-NAC	iTILs+sTILs	HE	NR	pCR	NR

**Table 2:** The evaluation of the risk of bias in research using the Newcastle–Ottawa scale

Study	Selection (0–4)				Comparability (0–2)		Outcome (0–3)			Total
	REC	SNEC	AE	DO	SC	AF	AO	FU	AFU	
Hwang, Hye Won et al. [11]	1	0	1	1	1	1	1	1	1	8
Ahn, S. G et al. [12]	1	0	1	1	1	0	1	0	0	5
Yang, Xia et al. [13]	1	0	1	1	1	0	1	1	1	7
Herrero-Vicent, C et al. [14]	0	0	1	1	0	0	1	1	0	4
Luen, S. J et al. [15]	1	0	1	1	1	0	1	1	1	7
Fujimoto, Yukie et al. [16]	1	0	1	1	1	1	0	1	1	7
Adams, S et al. [17]	1	1	1	1	1	1	1	1	1	9
Dieci, M. V et al.2014 [18]	1	0	1	1	1	1	0	1	1	7
Perez, E. A et al. [19]	1	0	1	1	1	1	1	1	1	8
Dieci, M. V et al.2015 [20]	1	0	1	1	1	1	1	1	1	8
Loi, S et al.2013 [5]	1	1	1	1	1	1	1	1	1	9
Loi, S et al.2014 [21]	1	1	1	1	1	1	1	1	1	9
Yasmin Issa-Nummer et al. [22]	1	0	1	1	1	1	1	1	1	8
Denkert, C et al.2010 [4]	1	1	1	1	1	1	1	1	1	9
Denkert, C et al.2015 [23]	1	1	1	1	1	1	1	1	1	9
Pruneri, G et al. [24]	1	0	1	1	1	1	1	1	1	8
Ingold Heppner, B et al. [25]	1	0	1	1	1	1	1	1	1	8
Denkert, C et al.2018 [26]	1	1	1	1	1	1	1	1	1	9
Wang, Qiong et al. [27]	0	0	1	1	1	0	0	1	1	5
Brodsky, Alexander S et al. [28]	0	0	1	1	1	0	0	1	1	5
Leon-Ferre, Roberto A et al. [29]	1	1	1	1	1	1	1	1	1	9
Salgado, Roberto et al. [30]	1	1	1	1	1	1	1	1	1	9
Ignatiadis, Michail et al. [31]	1	0	1	1	1	0	1	0	1	6
Mori, H et al. [32]	1	0	1	1	1	0	1	1	1	7
Dieci, M. V et al.2016 [33]	1	1	1	1	1	1	1	0	0	7
Ruan, Miao et al. [34]	1	0	1	1	1	0	1	0	0	5
O’Loughlin, Mark et al. [35]	1	0	1	1	1	0	1	0	0	5
Ali, H. Raza et al. [36]	1	1	1	1	1	1	1	0	0	7
Song, I. H et al. [37]	1	0	1	1	1	1	1	0	0	6
Li, X et al. [38]	1	0	1	1	1	1	1	0	0	6
Würfel, F et al. [39]	1	0	1	1	1	1	1	0	0	6
Hamy, A. S et al. [40]	1	1	1	1	1	1	1	0	0	7
Khoury, T et al. [41]	1	1	1	1	1	1	1	0	0	7

*REC* Representativeness of the exposed cohort, *SNEC* Selection of the non exposed cohort, *AE* Ascertainment of exposure, *DO* Demonstration that outcome of interest was not present at start of study, *SC* study controls for age, sex, *AF* study controls for any additional factor, *AO* Assessment of outcome, *FU* follow-up long enough for outcomes to occur (36 Months), *AFU* Adequacy of follow up of cohorts (≥90%).“1” means that the study is meeted the item and “0” means the opposite situation

### Relationship of lymphocyte-predominant breast cancer (LPBC) with clinicopathological parameters

#### T stage

The incidence of LPBC in the T3 and T4 groups was lower than that in the T1 and T2 groups, and the difference was statistically significant (OR = 0.646, 95% CI (0.542, 0.771), I^2^ = 0.0%, z = 4.85, *p* < 0.001). After that, subgroup analyses were conducted based on different countries [Europe: OR = 0.661, 95% CI (0.546, 0.800), I^2^ = 0.0%, z = 4.25, *p <* 0.001; Asia: OR = 0.516, 95% CI (0.297, 0.898), I^2^ = 0.0%, z = 2.34, *p* = 0.019; America: OR = 0.695, 95% CI (0.294, 1.643), z = 0.83, *p* = 0.407] and different study designs [randomized controlled trials (RCTs): OR = 0.663, 95% CI (0.550, 0.798), I^2^ = 0.0%, z = 4.33, *p* < 0.001; retrospective studies: OR = 0.516, 95% CI (0.297, 0.898), I^2^ = 0.0%, z = 2.34, *p* = 0.019]. In the Asia and Europe groups, the differences were statistically significant.

#### Lymph node status

The pooled analysis indicated that the incidence of LPBC detection between the lymph node metastasis group and the non-lymph node metastasis group was not significantly different (overall: OR = 0.941, 95% CI [0.681, 1.298], I^2^ = 76.4%, z = 0.37, *p* = 0.709). After that, subgroup analyses were carried out based on different countries [Europe: OR = 0.991, 95% CI (0.633, 1.551), I^2^ = 80.8%, z = 0.04, *p* = 0.968; Asia: OR = 1.013, 95% CI (0.595, 1.726), I^2^ = 60.8%, z = 0.05, *p* = 0.962; America: OR = 0.549, 95% CI (0.322, 0.936), z = 2.20, *p* = 0.028]. The difference was statistically significant in the America group.

#### Histological type

The incidence of LPBC was significantly different between the invasive ductal carcinoma and invasive lobular carcinoma groups (overall: OR = 2.654, 95% CI [1.132, 6.223], I^2^ = 68.0%, z = 2.24, *p* = 0.025). Then, subgroup analyses were performed based on different study designs (RCTs: OR = 4.735, 95% CI [2.850, 7.867], I^2^ = 0.0%, z = 6.00, *p* < 0.001; retrospective studies: OR = 1.101, 95% CI [0.622, 1.951], I^2^ = 0.0%, z = 0.33, *p* = 0.740). The difference was statistically significant in the RCT group.

#### Histological grade

The detection of LPBC in pathological specimens showed significant differences based on histological grading [III versus II and I, overall: OR = 2.889, 95% CI (2.218, 3.762), I^2^ = 49.5%, z = 7.87, *p* < 0.001]. After that, subgroup analyses were conducted based on different countries [Europe: OR = 2.871, 95% CI (2.290, 3.600), I^2^ = 25.5%, z = 9.14, *p* < 0.001; Asia: OR = 5.636, 95% CI (3.050, 10.415), I^2^ = 0.0%, z = 5.52, *p <* 0.001; America: OR = 1.659, 95% CI (0.982, 2.804), z = 1.89, *p* = 0.059] and different study designs [RCTs: OR = 2.763, 95% CI (2.188, 3.489), I^2^ = 39.7%, z = 8.53, *p <* 0.001; retrospective studies: OR = 3.284, 95% CI (1.359, 7.934), I^2^ = 64.0%, z = 2.64, *p* = 0.008]. In the Asia and Europe groups, the differences were statistically significant.

#### ER, PR and HER2 expression

The LPBC incidence rate in the ER+ group was significantly lower than that in the ER- group [total: OR = 0.291, 95% CI (0.185, 0.458), I^2^ = 70.0%, z = 5.35, *p* < 0.001]. After that, subgroup analyses were conducted based on different countries [Europe: OR = 0.348, 95% CI (0.197, 0.614), I^2^ = 61.1%, z = 3.65, *p* < 0.001; Asia: OR = 0.154, 95% CI (0.090, 0.264), z = 6.80, *p* < 0.001; America: OR = 0.342, 95% CI (0.216, 0.540), z = 4.60, *p <* 0.001] and different study designs [RCTs: OR = 0.360, 95% CI (0.230, 0.563), I^2^ = 60.1%, z = 4.49, *p <* 0.001; retrospective studies: OR = 0.191, 95% CI (0.105, 0.346), I^2^ = 30.4%, z = 5.44, *p <* 0.001]. In addition, PR+ and PR- groups were assessed [total: OR = 0.396, 95% CI (0.173, 0.906), I^2^ = 0.0%, z = 2.19, *p* = 0.028]. Furthermore, the detection rate of LPBC between the HER2+ group and the HER2- group was not significantly different [total: OR = 1.359, 95% CI (0.646, 2.858), z = 0.81, *p* = 0.419] and in subgroups based on different countries [Europe: OR = 1.443, 95% CI (0.529, 3.933), I^2^ = 92.0%, z = 0.72, *p* = 0.474; Asia: OR = 1.097, 95% CI (0.539, 2.230), z = 0.25, *p* = 0.799].

#### Ki-67 status

The incidence of LPBC was significantly different between the high Ki-67 and low Ki-67 groups (overall: OR = 6.378, 95% CI [3.674, 11.073], I^2^ = 30.1%, z = 6.58, *p* < 0.001).

#### Menopausal status

The LPBC detection rate between the premenopausal group and the postmenopausal group was not significantly different [total: OR = 0.963, 95% CI (0.716, 1.296), I^2^ = 0.0%, z = 0.25, *p* = 0.804]. After that, subgroup analyses were conducted based on different countries [Asia: OR = 1.036, 95% CI (0.629, 1.708), I^2^ = 29.3%, z = 0.14, *p* = 0.888; America: OR = 0.874, 95% CI (0.571, 1.339), z = 0.62, *p* = 0.537].

#### TNM stage

The LPBC detection rate between the stage III and IV group and the stage I and II group was not significantly different [total: OR = 0.825, 95% CI (0.220, 3.095), I^2^ = 81.4%, z = 0.29, *p* = 0.775]. After that, subgroup analyses were conducted based on country [Europe: OR = 0.431, 95% CI (0.211, 0.881), I^2^ = 0.0%, z = 2.31, *p* = 0.021; Asia: OR = 1.268, 95% CI (0.684, 4.050), I^2^ = 0.0%, z = 1.12, *p* = 0.261]. The difference was statistically significant in the Europe group. The results of the pooled analysis are summarized in Table 3.

**Table 3:** Detailed subgroup analysis of clinicopathological parameters

clinicopathological parameters	Different country				Different study design	
	Any	Europe	Asia	America	RCT	Retrospective
Age > 50 vs. ≤ 50 (OR)	0.873 [0.761,1.002]; I2 = 0.0%; z = 1.93; *p* = 0.054	0.868 [0.754,1.000]; I2 = 0.0%; z = 1.96; *p* = 0.049	0.990 [0.513,1.912]; z = 0.03; *p* = 0.976	_	0.869 [0.753,1.002]; I2 = 0.0%; z = 1.93; *p* = 0.054	0.935 [0.563, 1.554]; I2 = 0.0%; z = 0.26; *p* = 0.795
pT:T3/T4 vs. T1/T2 (OR)	0.646 [0.542,0.771]; I2 = 0.0%%; z = 4.85; *p* < 0.001	0.661 [0.546,0.800]; I2 = 0.0%; z = 4.25; *p* < 0.001	0.516 [0.297,0.898]; I2 = 0.0%; z = 2.34; *p =* 0.019	0.695 [0.294,1.643]; z = 0.83; *p =* 0.407	0.663 [0.550,0.798]; I2 = 0.0%; z = 4.33; *p* < 0.001	0.516 [0.297, 0.898]; I2 = 0.0%; z = 2.34; *p =* 0.019
LN(+) vs. LN(−)(OR)	0.941 [0.681,1.298]; I2 = 76.4%; z = 0.37; *p* = 0.709	0.991 [0.633,1.551]; I2 = 80.8%; z = 0.04; *p* = 0.968	1.013 [0.595,1.726]; I2 = 60.8%; z = 0.05; *p* = 0.962	0.549 [0.322,0.936]; z = 2.20; *p* = 0.028	1.003 [0.651,1.546]; I2 = 82.4%; z = 0.01; *p* = 0.989	0.858 [0.501,1.468]; I2 = 66.5%; z = 0.56; *p* = 0.576
IDC vs. ILC(OR)	2.654 [1.132,6.223]; I2 = 68.0%; z = 2.24; *p =* 0.025	2.642 [0.700,9.967]; I2 = 84.0%; z = 1.43; *p* = 0.152	2.883 [0.766,10.85]; I2 = 0.0%; z = 1.57; *p* = 0.118	2.571 [0.614,10.77]; z = 1.29; *p* = 0.196	4.735 [2.850,7.867]; I2 = 0.0%; z = 6.00; *p* < 0.001	1.101 [0.622,1.951]; I2 = 0.0%; z = 0.33; *p* = 0.740
Histological grade:III vs.I–II(OR)	2.889 [2.218,3.762]; I2 = 49.5%; z = 7.87; *p <* 0.001	2.871 [2.290,3.600]; I2 = 25.5%; z = 9.14; *p <* 0.001	5.636 [3.050,10.42]; I2 = 0.0%; z = 5.52; *p <* 0.001	1.659 [0.982,2.804]; z = 1.89; *p* = 0.059	2.763 [2.188,3.489]; I2 = 39.7%; z = 8.53; *p <* 0.001	3.284 [1.359,7.934]; I2 = 64.0%; z = 2.64; *p* = 0.008
ER (+) vs.(−) (OR)	0.291 [0.185,0.458]; I2 = 70.0%; z = 5.35; *p <* 0.001	0.348 [0.197,0.614]; I2 = 61.1%; z = 3.65; *p <* 0.001	0.154 [0.090,0.264]; z = 6.80; *p <* 0.001	0.342 [0.216,0.540]; z = 4.60; *p <* 0.001	0.360 [0.230,0.563]; I2 = 60.1%;z = 4.49; *p <* 0.001	0.191 [0.105,0.346]; I2 = 30.4%; z = 5.44; *p <* 0.001
PR (+) vs.(−) (OR)	0.396 [0.173,0.906]; I2 = 0.0%; z = 2.19; *p =* 0.028	_	_	_	_	_
HER2 (+) vs.(−) (OR)	1.359 [0.646,2.858]; I2 = 88.0%; z = 0.81; *p* = 0.419	1.443 [0.529,3.933]; I2 = 92.0%; z = 0.72; *p* = 0.474	1.097 [0.539,2.230]; z = 0.25; *p* = 0.799	_	1.871 [0.486,7.205]; I2 = 95.9%;z = 0.91; *p* = 0.362	0.961 [0.544, 1.699]; I2 = 0.0%; z = 0.14; *p* = 0.891
Ki-67: high vs. low	6.378 [3.674,11.073]; I2 = 30.1%; z = 6.58; *p <* 0.001	_	_	_	_	_
premenopausal vs. postmenopausal	0.963 [0.716,1.296]; I2 = 0.0%; z = 0.25; *p =* 0.804	_	1.036 [0.629,1.708]; I2 = 29.3%; z = 0.14; *p =* 0.888	0.874 [0.571,1.339]; z = 0.62; *p =* 0.537	_	_
TNM stage: III, IV vs. I, II	0.825 [0.220,3.095]; I2 = 81.4%; z = 0.29; *p =* 0.775	0.431 [0.211,0.881]; I2 = 0.0%; z = 2.31; *p =* 0.021	1.268 [0.684,4.050]; I2 = 0.0%; z = 1.12; *p =* 0.261	_	_	_

#### Impact of TILs on pCR

**Fig. 2:**
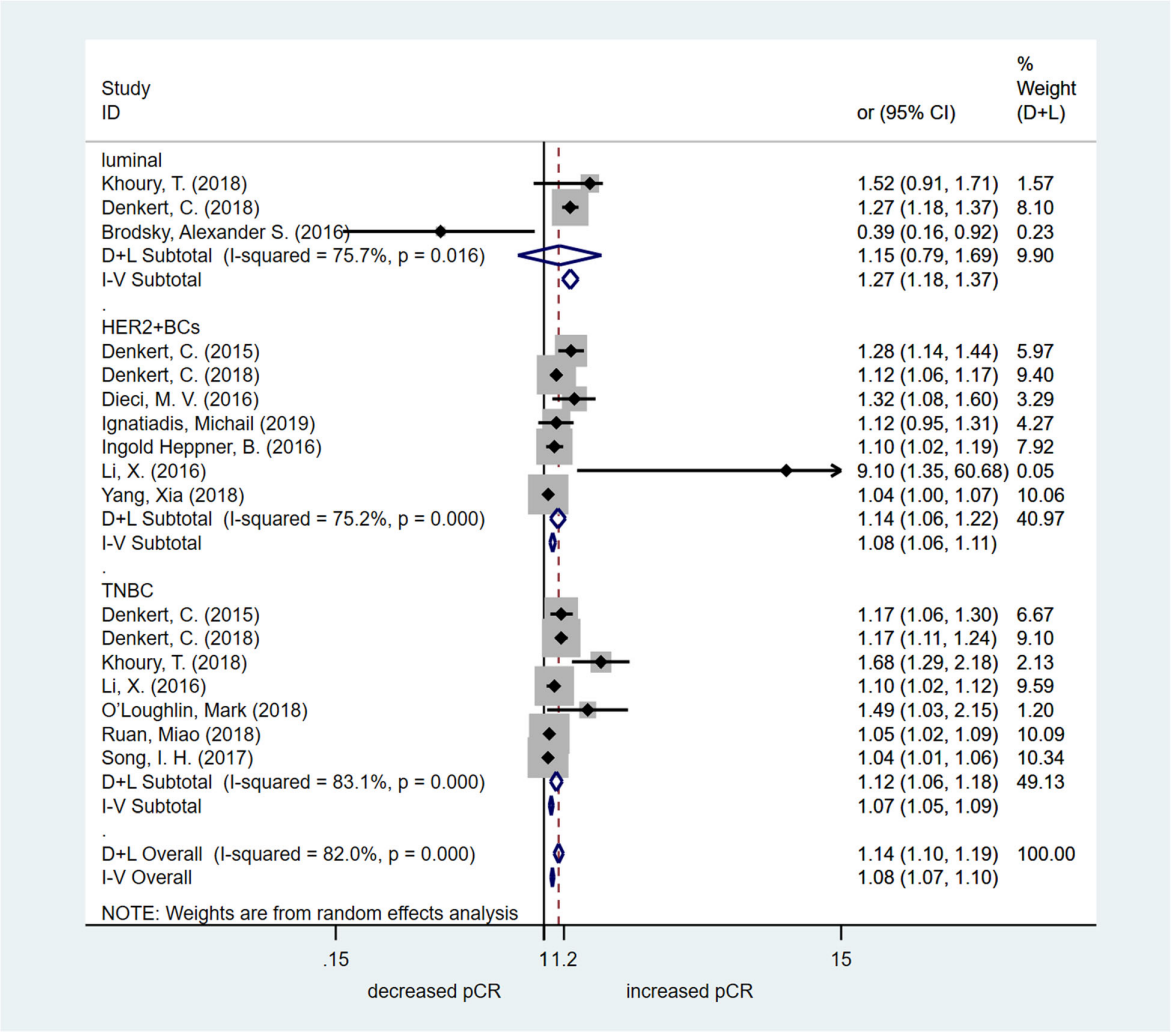
Forest plot of OR for pCR. Pooled assessing OR for pCR

To further assess the predictive effect of TIL detection in breast cancer patients with different molecular subtypes, the OR value of pCR was analysed by meta-analysis. In this meta-analysis, we chose studies that focused on TILs as a continuous parameter (per 10% increments). The OR value of pCR was available in three studies including the luminal molecular subtype of breast cancer. There was no significant increase in the pCR rate in the high TIL group [OR = 1.154, 95% CI (0.789–1.690), *p* = 0.460]. The OR value of pCR was available in seven studies including the HER2-enriched molecular subtype of breast cancer. The assessed pooled OR value confirmed that high TILs were associated with significantly increased pCR rates [OR = 1.137, 95% CI (1.061–1.218), *p* < 0.001]. The OR value of pCR was available in seven studies including the triple-negative breast cancer (TNBC) molecular subtype. The estimated pooled OR value showed that high TILs were associated with significantly increased pCR rates [OR = 1.120, 95% CI (1.061–1.182), *p* < 0.001]. The OR value of pCR was available in nine studies including all breast cancer patients. The assessed pooled OR value confirmed that high TILs were associated with significantly increased pCR rates [OR = 1.214, 95% CI (1.108–1.329), *p* < 0.001]. High-quality studies (NOS score > 6) were used to perform the sensitivity analysis, and the results were consistent (HER2-enriched molecular subtype of breast cancer: OR = 1.133, 95% CI [1.057–1.215], *p* < 0.001; TNBC molecular subtype: OR = 1.237, 95% CI [1.094–1.399], *p* = 0.001). However, in breast cancer patients with the luminal molecular subtype, the estimated pooled OR value showed that high TILs were associated with significantly increased pCR rates [OR = 1.298, 95% CI (1.157–1.456), *p <* 0.001]. Fig. 2 summarizes the results of the pCR assessment. Publication bias was detected by the funnel plot (Fig. 5a). Egger’s test indicated that there was publication bias.

#### Effect of TILs on prognosis (OS and DFS)

**Fig. 3:**
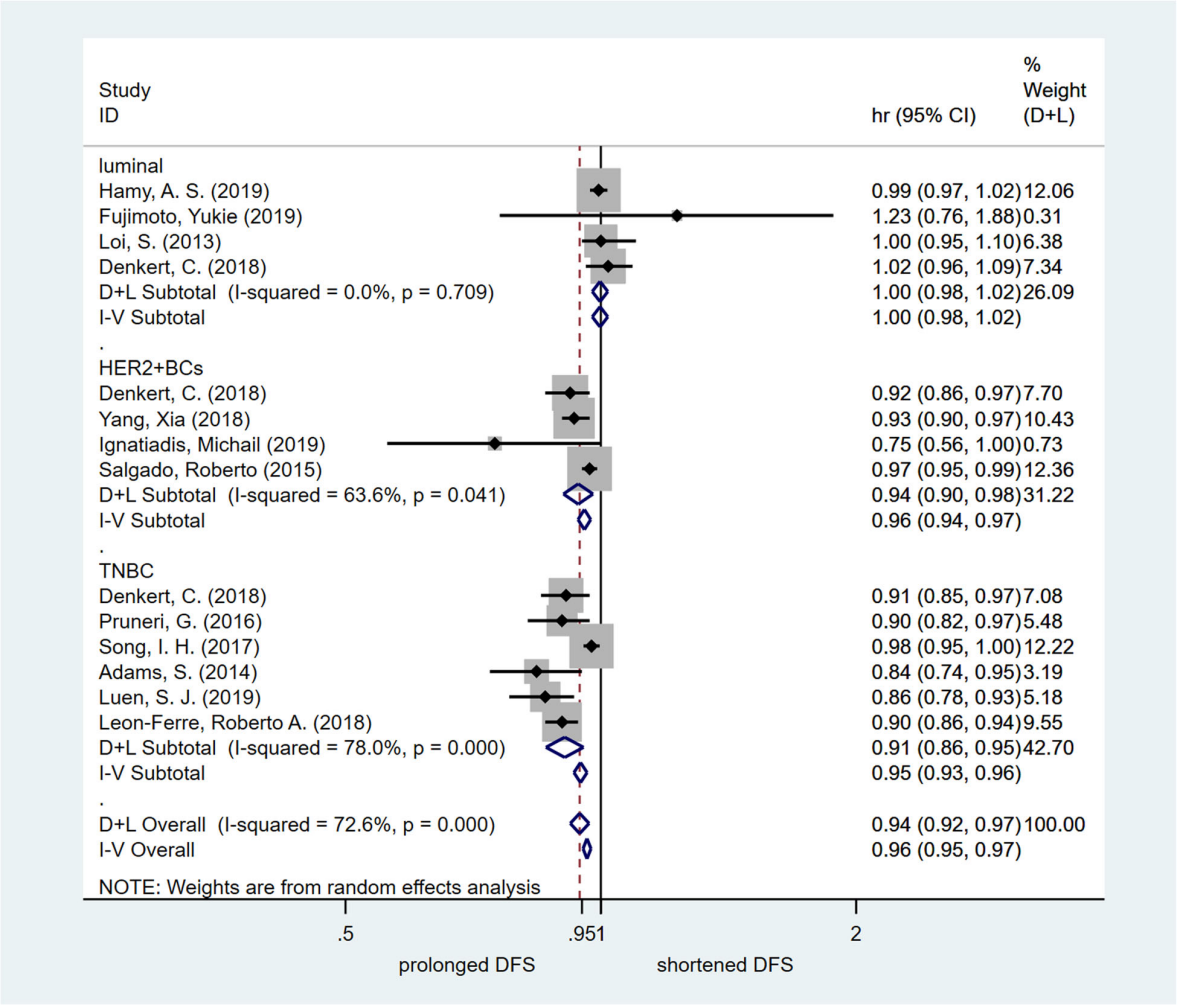
Forest plot of HR for DFS. Pooled assessing HR for DFS

To further estimate the survival impact of TIL detection in breast cancer patients with different molecular subtypes, the HR value of DFS or OS was analysed by meta-analysis. In this meta-analysis, we chose studies that focused on TILs as a continuous parameter (per 10% increments). Four studies on the luminal molecular subtype of breast cancer provided HR values for DFS. There was no significant improvement in DFS in the high TIL group [HR = 0.998, 95% CI (0.977–1.019), *p* = 0.840]. Four studies on the HER2-enriched molecular subtype of breast cancer provided HR values for DFS. The assessed pooled HR values confirmed that high TILs were associated with significantly increased DFS [HR = 0.940, 95% CI (0.903–0.979), *p* = 0.003]. Six studies on the TNBC molecular subtype provided HR values for DFS. The estimated pooled HR value showed that high TILs were associated with significantly increased DFS [HR = 0.907, 95% CI (0.862–0.954), *p* < 0.001]. Four studies of all breast cancer patients provided HR values for DFS. The assessed pooled HR values confirmed that high TILs were associated with significantly increased DFS [HR = 0.988, 95% CI (0.979–0.997), *p* = 0.012]. High-quality studies (NOS score > 6) were used to carry out the sensitivity analysis, and the results were consistent (breast cancer with the HER2-enriched molecular subtype: HR = 0.946, 95% CI [0.913 ~ 0.980], *p* = 0.002; TNBC molecular subtype: HR = 0.893, 95% CI [0.867 ~ 0.921], *p* < 0.001; breast cancer with the luminal molecular subtype: HR = 0.998, 95% CI [0.977 ~ 1.019], *p* = 0.840). Fig. 3 summarizes the results of the DFS assessment. Publication bias was detected by the funnel plot. No significant publication bias was found (Fig. 5b). Egger’s test indicated that there was not publication bias.

**Fig. 4:**
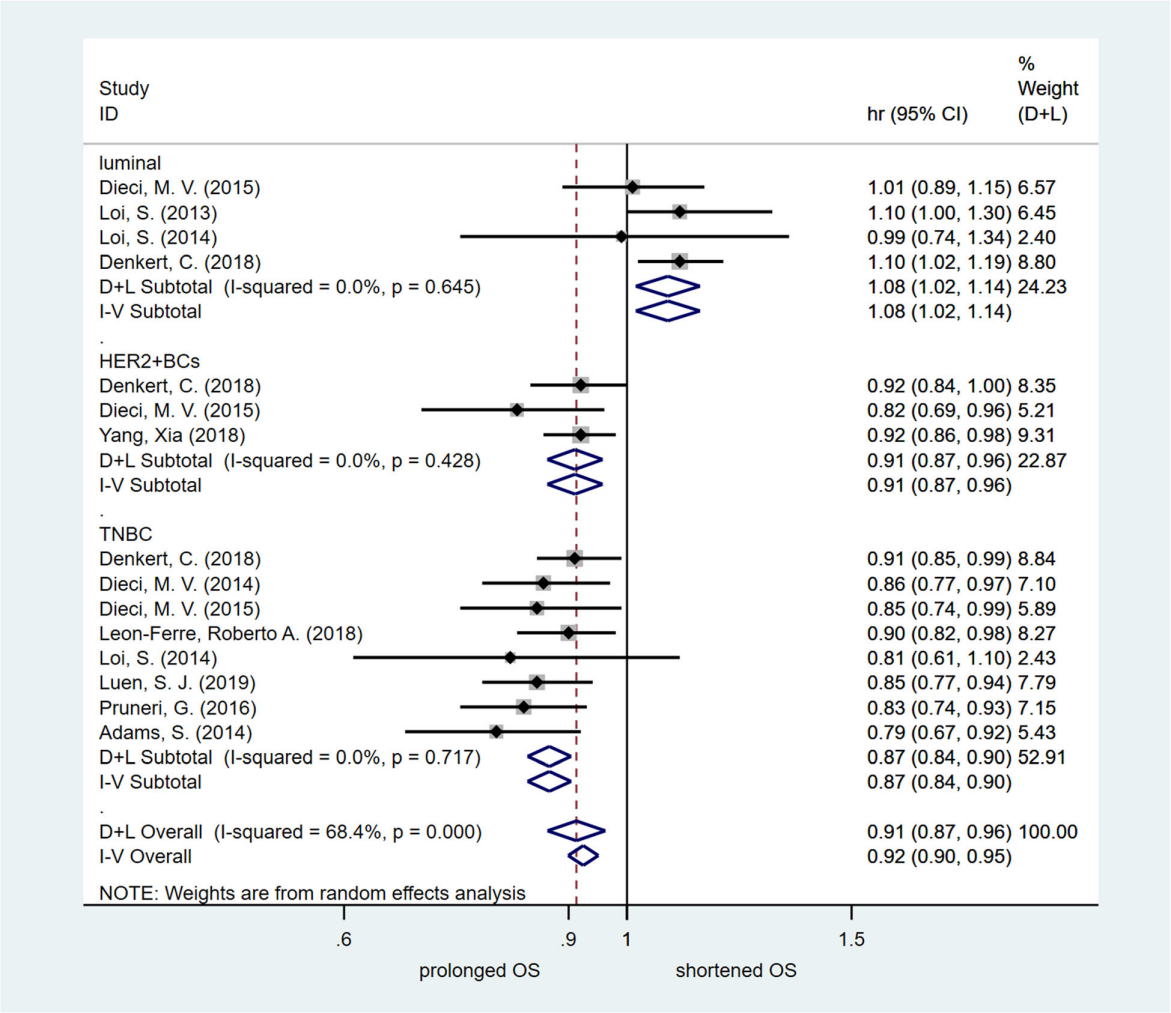
Forest plot of HR for OS. Pooled assessing HR for OS

**Fig. 5:**
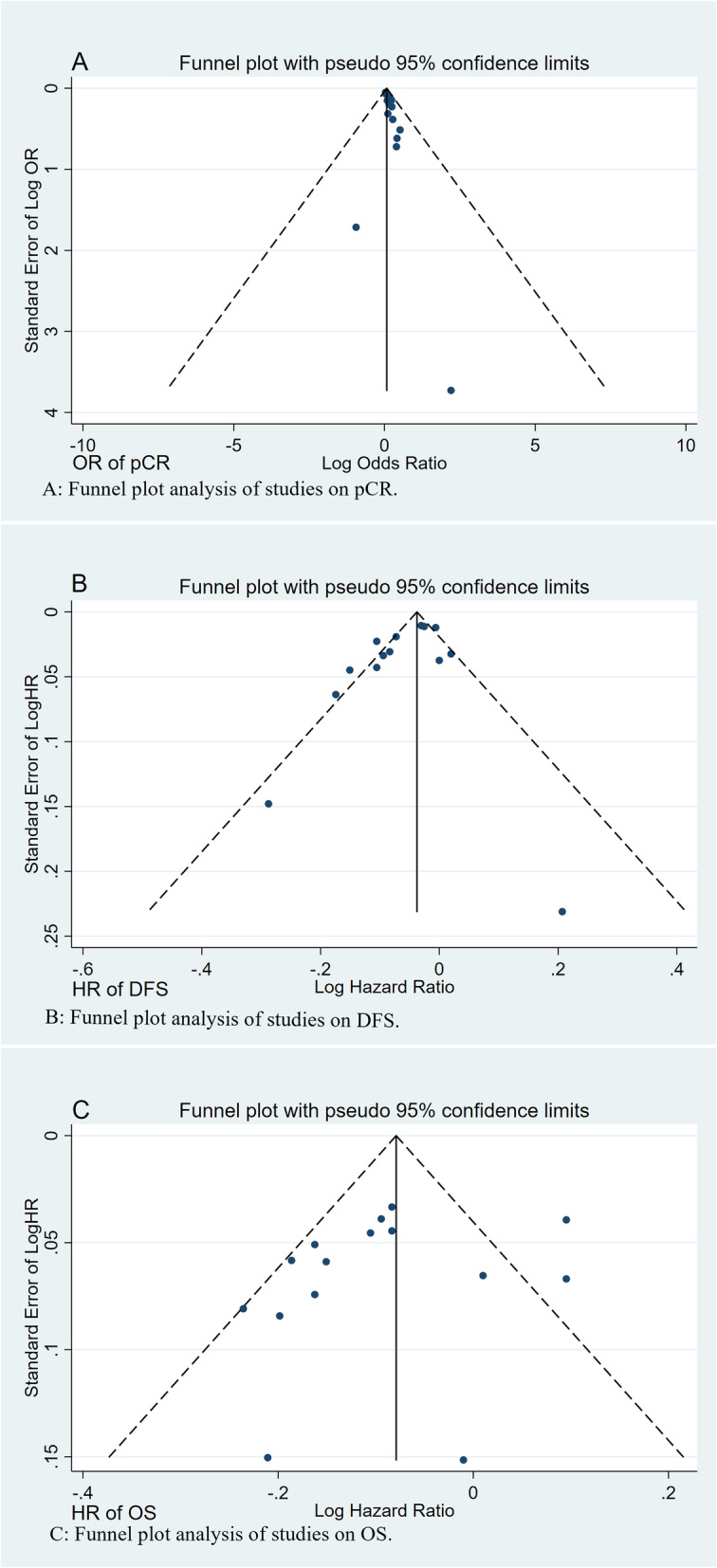
Funnel plot for potential publication bias

In addition, the HR values of OS were obtained in four studies. The pooled analysis confirmed that the high TIL group of the luminal molecular subtype of breast cancer was significantly associated with unfavourable OS [HR = 1.077, 95% CI (1.016 ~ 1.141), *p* = 0.012]. In contrast, the HR values of OS were obtained in three studies on patients with the HER2-enriched molecular subtype of breast cancer. The assessed pooled HR values showed that high TILs were associated with significantly favourable OS [HR = 0.910, 95% CI (0.866–0.957), *p* < 0.001]. The HR values of OS were obtained in eight studies on patients with the TNBC molecular subtype. The evaluated pooled HR values indicated that high TILs were associated with significantly favourable OS [HR = 0.869, 95% CI (0.836 ~ 0.904), *p <* 0.001]. The HR values of OS were obtained in four studies of all breast cancer patients. The estimated pooled HR value confirmed that high TILs were not associated with significantly favourable OS [HR = 1.017, 95% CI (0.983–1.052), *p* = 0.324]. High-quality studies (NOS score > 6) were used to conduct the sensitivity analysis, and the results were consistent. Fig. 4 summarizes the results of the OS assessment. Publication bias was tested by the funnel plot. No significant publication bias was found (Fig. 5c). Egger’s test indicated that there was not publication bias.

## Discussion

Breast cancer is a highly heterogeneous disease in terms of its clinical processes and molecular types. At present, standardized systemic therapy has significantly increased the survival of breast cancer patients, but metastasis and recurrence remain the determinants of death. Therefore, how to further reduce recurrence and metastasis is still a key issue in clinical practice. The complex interaction between the immune system and cancer cells plays a vital role in controlling and eradicating cancer [3]. A few decades ago, people noticed that the tumour microenvironment contained a variable number of lymphocytes, later called tumour-infiltrating lymphocytes or TILs [43]. TILs have become a potential biomarker for survival prediction in breast cancer patients [4, 5]. In patients with different molecular subtypes, a comprehensive evaluation of the clinical impact of TILs will help to uncover the important mechanisms of the interaction between tumour and host immunity. Nevertheless, the clinical significance of TILs in patients with different molecular subtypes is still unclear. By summarizing and analysing relevant high-quality studies, our meta-analysis aims to provide evidence for determining the clinical significance of TILs in the different molecular subtypes of breast cancer.

The pooled analysis confirmed that LPBC was significantly correlated with higher histopathological grade. Moreover, our meta-analysis indicated that LPBC was related to Ki-67, ER and PR status. Afterwards, sensitivity analysis excluding low-quality studies showed consistent results. Whether TILs play different roles in patients with different molecular subtypes remains unknown. We further analysed the prognostic value and predictive roles of TILs in patients with different molecular subtypes. To further estimate the survival impact of TIL detection in patients with different molecular subtypes, the HR values of DFS and OS were analysed by meta-analysis. The assessed pooled OR value confirmed that high TILs were correlated with significantly increased pCR rates in patients with the HER2-enriched molecular subtype of breast cancer in multivariate analysis studies. The assessed pooled HR values confirmed that high TILs were correlated with significantly increased DFS. The assessed pooled HR values showed that high TILs were related to significantly favourable OS. The sensitivity analysis showed the robustness of the HR estimates.

For the TNBC molecular subtype, the estimated pooled OR value showed that high TILs were related to significantly improved pCR rates in multivariate analysis studies. Furthermore, the assessed pooled HR values confirmed that high TILs were correlated with significantly improved DFS and favourable OS in multivariate analysis studies.

For the luminal molecular subtype of breast cancer, there was no significant increase in the pCR rate in the high TIL group. In addition, there was no significant improvement in DFS in the high TIL group. Conversely, the pooled analysis confirmed that the high TIL group of the luminal molecular subtype of breast cancer was significantly correlated with unfavourable OS. Considering the small number of studies, the results of this analysis should be interpreted with caution.

Our meta-analysis confirmed that TILs are an ideal biomarker for TNBC and the HER2-enriched molecular subtype of breast cancer in the prediction of pCR and favourable prognosis. In contrast, TILs are a biomarker for predicting poor OS in the luminal molecular subtype of breast cancer. Therefore, TILs should be monitored in breast cancer patients for rational stratification and adjustment of the treatment strategy, and further detailed and in-depth studies on TILs and breast cancers of different molecular subtypes are needed. Further study on the different roles of different TIL subclasses in the different molecular subtypes of breast cancer will help us further understand the precise mechanisms of TILs and provide more evidence for the immunotherapy of breast cancer with different molecular subtypes.

The limitations of this meta-analysis include the following aspects. First, heterogeneity cannot be avoided completely, so we chose a random effect model. Second, fewer high-quality stratified studies on the different molecular subtypes of breast cancer can affect the statistical efficacy of our results. Therefore, it is necessary to conduct more prospective clinical studies to clarify the true usefulness of TILs. Third, our study was based on data provided by different studies, not individual patient data, so reliable correlation estimates could not be made. Although our research has some limitations, we systematically evaluated a large number of high-quality studies with multivariate analysis, and the research results may be a reliable reference for guiding clinical practice.

## Conclusions

In conclusion, we performed a meta-analysis including thirty-three high-quality studies that implemented multivariate analysis, and 18,170 patients with different molecular subtypes of breast cancer were analysed. Our meta-analysis confirms that high TILs are correlated with favourable survival and predict pCR in breast cancer patients with TNBC and the HER2-enriched molecular subtype. Conversely, the pooled analysis confirmed that the high TIL group of the luminal molecular subtype of breast cancer was significantly correlated with unfavourable OS. Large-scale, multicentre and well-designed high-quality studies are needed to study the role of different TIL subclasses in the different molecular subtypes of breast cancer. Moreover, it can provide guidance for the clinical practice of breast cancer with different molecular subtypes.

## Data Availability

All data generated or analysed during this study are included in this published article [and its supplementary information files].

## Abbreviations

TILs: Tumor-infiltrating lymphocytes
sTILs: Stromal tumor-infiltrating lymphocytes
iTILs: Intratumoral tumor-infiltrating lymphocytes
OR: Odds ratio
HR: Hazard ratio
CI: Confidence intervals
pCR: Pathologic complete response
DFS: Disease-free survival
OS: Overall survival
RFS: Relapse-free survival
BCSS: Breast cancer-specific survival
DRFI: Distant recurrence–free interval
MFS: Metastases-free survival
DDFS: Distant disease-free survival
BCFI: Breast cancer-free interval
EFS: Event-free survival
HE: Hematoxilin and eosin
IHC: Immunohistochemistry
CNB: Core needle biopsy
NAC: Neoadjuvant chemotherapy
CRT: Chemoradiotherapy
TMA: Tissue microarrays
B-NC: Before Neoadjuvant chemotherapy
post: Postoperative
NR: Not reported
LN: Lymph node
ER: Estrogen receptor
PR: Progesterone receptor
HER2: Human epidermal growth factor receptor-2
TNBC: Triple-negative breast cancer
LPBC: Lymphocyte-predominant breast cancer
RCT: Randomized controlled trial
NOS: Newcastle-Ottawa Scale
